# A Dynamic Approach to Compulsive Fantasy: Constraints and Creativity in “Maladaptive Daydreaming”

**DOI:** 10.3390/bs15101333

**Published:** 2025-09-28

**Authors:** Jennifer I. Burrell, Emily Lawson, Kalina Christoff Hadjiilieva

**Affiliations:** 1 Department of Psychology, University of British Columbia, Vancouver, BC V6T 1Z4, Canada; 2 Department of Philosophy, University of British Columbia, Vancouver, BC V6T 1Z1, Canada; emily.lawson@ubc.ca

**Keywords:** maladaptive daydreaming, compulsive fantasy, compulsion, fantasy, daydreaming, creativity, imagination, the Dynamic Framework of Thought, spontaneous thought, mind-wandering

## Abstract

Compulsive fantasy, often called “maladaptive daydreaming,” involves frequent engagement with immersive fantasies that can sometimes interfere with everyday life and cause distress. This paper expands on Christoff and colleagues’ Dynamic Framework of Thought (DFT) to offer a process-based analysis of compulsive fantasy as it relates to other mental phenomena such as daydreaming and creative thought. Drawing on the existing literature and posts on online forums by self-identified maladaptive daydreamers, we also propose an account of how compulsive fantasy episodes may unfold in terms of the oscillating dynamics of various constraints on thought, and how these dynamics may be related to a perceived struggle with agency. Automatic constraints, including affective salience and mental habits, may bring about a fantasy episode. During a fantasy episode, automatic constraints may be relatively high throughout, whereas deliberate constraints may be intermittently engaged to influence the fantasy. Our analysis supports the use of “compulsive fantasy” as a more accurate designation than “maladaptive daydreaming” for this phenomenon: compulsive fantasies are not daydreams, because they are more constrained in their mental dynamics. We show that fantasy and daydreaming are not inherently harmful but can become so when they are accompanied by relatively strong and sustained automatic constraints on thought.

## 1. Introduction

We are only beginning to uncover the diversity of human imagination. Empirical attention to the phenomenal character of thought is revealing new dimensions of inner life—for example, some subjects lack conscious mental imagery across a range of sensory modalities, while others describe experiencing mental imagery that is just as vivid and realistic as sensory perception (Milton et al., 2021). Recently, a growing body of research has identified a population of particularly immersive, vivid imaginers, many of whom devote significant time and care to crafting elaborate fantasy worlds that can, according to many self-reported “immersive daydreamers,” compete with the vivacity and allure of embodied life. Many of these extraordinary imaginers describe this practice as enriching their lives. Others, however, find that imaginative fantasies that feel more compelling than reality can pull them into a bewildering and painful struggle. When fantasy becomes compulsive, it can become what those who experience it and researchers describe as a form of addiction (e.g., Pietkiewicz et al., 2018). This mental behaviour is difficult for friends and family to detect because of its private nature and difficult for imaginers to resist because to engage in the addictive behaviour is no more difficult than thinking.

Imaginers who compulsively fantasize in this way often refer to themselves as “Maladaptive daydreamers” or “MDers” in online communities, following the uptake of early research on the phenomenon by Somer (2002). “Maladaptive daydreaming” refers to compulsive engagement with immersive fantasies. Many people who experience this phenomenon develop long-term, rich imaginative worlds containing emotionally charged storylines, often centred on a character or characters that function as idealized versions of themselves. These fantasies are described as extraordinarily vivid, detailed, and compelling, and fantasizers often use kinesthetic movements to focus attention on mental imagery and facilitate absorption (Bigelsen et al., 2016; Bigelsen & Schupak, 2011; Somer, 2002). This activity can become problematic when obsessive, irresistible urges to fantasize result in fantasy activity that consumes the majority of imaginers’ waking hours, thus interfering with everyday life and causing distress.

**Figure 1:**
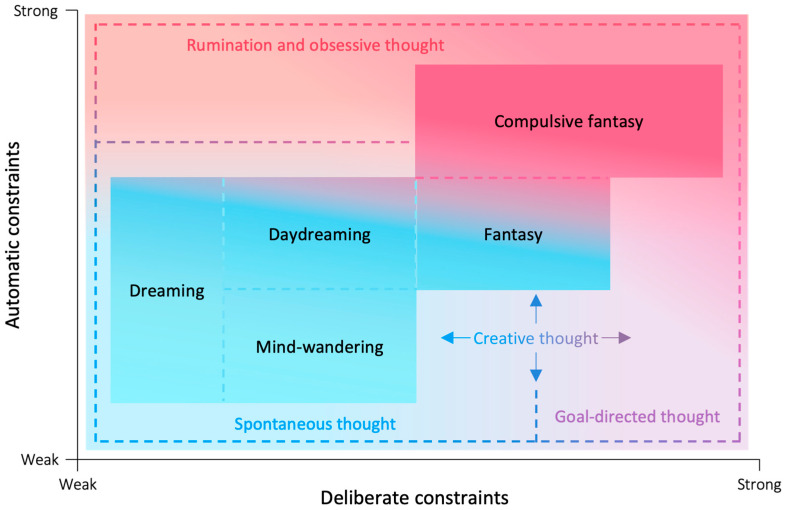
Daydreaming, fantasy, and compulsive fantasy conceptualized using the Dynamic Framework of Thought (Christoff et al., 2016). We propose that compulsive fantasy arises under the influence of strong levels of automatic and medium to high levels of deliberate constraints.

In this paper, we use “compulsive fantasy” as a more precise designation for this phenomenon. A shift towards the use of “compulsive fantasy” as a preferred term appears to be underway: Bigelsen and Schupak, responsible for a seminal study on the topic, acknowledge that “considerable confusion” has arisen from the term “maladaptive daydreaming” and recommend that the term “compulsive fantasy” be used in its stead, given that the phenomenon in question is “exclusive to definitions of fantasizing, and more specifically, of fantasizing of a compulsive nature” (Bigelsen & Schupak, 2011, pp. 1635–1636; Schupak & Rosenthal, 2009). Maladaptive daydreaming is now increasingly defined in terms of compulsive fantasy (e.g., Chefetz et al., 2023; Nowacki & Pyszkowska, 2024; Roy et al., 2024). The term “compulsive” captures the distinctive forms of struggle the behaviour involves and is preferable to “maladaptive,” not least because speculation about evolutionary or adaptive functions should not be of primary consideration in defining a cognitive phenomenon. The use of “compulsive” is also consistent with definitions in the literature on Obsessive–Compulsive Disorder. For example: “compulsions are urges that people have to do something to lessen feelings of anxiety or other discomfort” (Goodman et al., 1989).^1^ We also believe that the term “fantasy” is more appropriate than “daydreaming” because, as we argue here, these two terms refer to distinct phenomena. More specifically, compulsive fantasy differs from daydreaming by virtue of its dynamic profile (Figure 1). The goal of this paper is to provide a conceptual and theoretical foundation for future research on compulsive fantasy and its relationship to other forms of thought.

Reconceptualization is needed because research on compulsive fantasy is relatively new. Although “maladaptive daydreaming” has received little conceptual or philosophical attention, a number of researchers have urged its inclusion in the DSM-5 (Bigelsen et al., 2016; Pietkiewicz et al., 2018; Somer et al., 2017). We believe that this is premature and potentially detrimental, given the lack of conceptual clarity about this mental phenomenon. In the larger yet still emerging field of cognitive neuroscience of spontaneous thought (Christoff & Fox, 2018), terminology remains in flux: terms like “daydreaming,” “mind-wandering,” “fantasy,” and “imagination” are sometimes used interchangeably and still lack settled definitions in the literature. Until recently, these phenomena were primarily defined in relation to “task-independent” or “stimulus-independent” thought. Jerome Singer, the founder of daydreaming research (Singer, 1975, 1976), defined daydreaming broadly as any thought not directed at the immediate task. Singer and Antrobus’s “daydreaming frequency scale” defined daydreaming as “thoughts unrelated to a task you are working on” (Singer & Antrobus, 1970, p. 2). Later research continued this trend of emphasizing task-unrelatedness and stimulus-independence (e.g., Killingsworth & Gilbert, 2010; Klinger, 2009; Kucyi & Davis, 2014; McVay et al., 2009; Singer, 1976; Smallwood & Schooler, 2015; Stawarczyk et al., 2012; Teasdale et al., 1995; Zedelius et al., 2021) and introduced related concepts such as “nonworking thought” (Klinger, 1971) and “self-generated thought” (Andrews-Hanna et al., 2013). An unwarranted conceptual conflation between mind-wandering and daydreaming has been one of the negative consequences of defining both in terms of their task-unrelatedness and stimulus-independence, as argued in our previous paper by Lawson and Thompson (2024) and others (Dorsch, 2015). As we have argued elsewhere (Christoff et al., 2018), relying on task-relatedness alone lacks the precision necessary to guide future research on imagination and spontaneous thought.

The concepts of fantasy and imagination are subject to a similar definitional confusion. The two terms are often conflated (Regis, 2013; Person, 1995; Spector Person, 2003) and used to refer either to unconscious, wish-fulfilling drives posited by psychoanalysts (see Hayman, 1989) or to any “fanciful” or non-goal-oriented thought (Klinger, 1990, 2009). For example, according to Klinger, “Fantasy is defined as verbal reports of all mentation whose ideational products are not evaluated by the subject in terms of advancing some immediate goal extrinsic to the mentation itself” (Klinger, 1971, pp. 9–10). An exclusive focus on thought content and task independence leaves many questions about thought unanswered and neglects the temporal dynamics of thought processes. In contrast, attending to thought dynamics, as we do in this paper, can help to “cut through the definitional haze” (Christoff et al., 2018). For example, Lawson and Thompson (2024) use thought dynamics to argue that while daydreaming and mind-wandering are both forms of spontaneous thought, daydreaming is distinguished from mind-wandering by its dreamlike, immersive, imaginative quality, and we accept this more precise definition of daydreaming as “spontaneous immersive imagination in the waking state” here.

Theoretical work can help refine our conceptual and empirical understanding of the differences between “daydreaming,” “fantasy,” and “compulsive fantasy.” Different forms of thought, such as mind-wandering, creative thought, and rumination, can be distinguished based on their constraints, as described by Christoff and colleagues’ Dynamic Framework of Thought (DFT, Christoff et al., 2016). According to the DFT, thoughts arise under two main types of constraints—automatic and deliberate constraints—of varying strengths. Identifying where different mental experiences fall on these continua can help to specify and distinguish various forms of thought (Figure 1).

*Deliberate constraints* on thought are flexible and implemented through cognitive control. For example, intentionally directing one’s attention to solving a problem is a way of imposing a deliberate constraint on thought. Varying strengths and dynamics of deliberate constraints can help distinguish spontaneous thought and creative thought, for example: while spontaneous thought (including mind-wandering and daydreaming) proceeds in the context of relatively low deliberate control, creative thought processes tend to oscillate between spontaneous generation and deliberate evaluation of ideas (Christoff et al., 2016; Ellamil et al., 2012; Girn et al., 2020; Zamani et al., 2022).

*Automatic constraints* on thought are a family of mechanisms that also limit the contents of thoughts and how they change over time but operate outside of cognitive control. For example, moods, habits, and the salience of exteroceptive events, such as a dog’s bark or a fly’s buzz, may influence and limit what we think about. Similarly, salient interoceptive sensations such as intense hunger may automatically limit one’s thoughts to food. Because automatic constraints can operate outside of the thinker’s explicit awareness, the experience of automatic constraints may be less phenomenologically obvious than that of deliberate cognitive control. Automatic constraints may become more apparent, however, when they run counter to deliberate intentions. The experience of automatic constraints may be characterized by the feeling of thoughts being pulled in a certain direction, regardless of the intended direction of the thoughts. For example, thoughts about food might pull one’s attention from the work one intends to focus on. The level of automatic constraints can be used to distinguish between forms of thought, such as mind-wandering and rumination. Mind-wandering and daydreaming thoughts flow from one topic to the next relatively freely, under relatively low automatic constraints (Christoff et al., 2016; Lawson & Thompson, 2024). Ruminative thoughts, by contrast, are pulled back to the same contents repeatedly under pressures from strong automatic constraints (Christoff et al., 2016) or guidance (Irving, 2016; Irving & Thompson, 2018). Here, we use the DFT to analyze the experience of compulsive fantasy and distinguish it conceptually and phenomenologically from related mental experiences (Figure 1).

In the remainder of this paper, we offer a process-based analysis of compulsive fantasy, and we propose how compulsive fantasy episodes may unfold in terms of the oscillating dynamics of various constraints on thought. Section 2 offers an account of fantasy as a mental activity that can be distinguished from daydreaming. Section 3 and Section 4 propose a dynamic account of how automatic and deliberate constraints may operate during the onset of fantasy episodes. Section 5 describes how automatic and deliberate constraints may interact and oscillate across the unfolding of a fantasy episode, and Section 6 compares these oscillatory dynamics to similar patterns that may occur in creative thought.

Overall, this paper offers an expanded treatment of the DFT to include a conceptual analysis of compulsive fantasy and its relationship to other mental phenomena, such as daydreaming, mind-wandering, and creative thought. Analyzing compulsive fantasy through this expansion of the DFT offers strong support in favour of using the term “compulsive fantasy” in place of the potentially misinformative term “maladaptive daydreaming.” We also provide an account of how compulsive fantasy episodes may unfold in terms of the oscillating dynamics of various constraints on thought. The picture that emerges is one of struggle with agency: fantasies emerge and unfold under complex and shifting pressures. We propose that strong automatic and deliberate constraints likely guide the onset of compulsive fantasy episodes, while the unfolding of the episode itself involves patterns of constraints similar to those that occur during creative thought processes, but with relatively high levels of automatic constraints throughout the fantasy episode.

## 2. Conceptualizing Fantasy and Its Relationship to Daydreaming

Before turning to the dynamics of compulsive fantasy, we offer a brief conceptual formulation of fantasy. We characterize fantasies as emotionally gratifying, narrative-driven, imaginative projects that often involve vicariously sharing feelings with proxy characters. Fantasizing and daydreaming have often been treated as synonymous (e.g., Regis, 2013), in part because both activities are imaginative. Indeed, daydreaming and fantasizing are closely connected activities on a multi-dimensional spectrum of thought (Figure 1). Fantasy is closely related to other waking imaginative experiences, including daydreaming. However, differences between these activities are important with respect to conceptualizing compulsive fantasy.

Daydreaming can be conceptualized as spontaneous, immersive imagination in the waking state (Lawson & Thompson, 2024). Like mind-wandering and other related forms of spontaneous thought, daydreaming proceeds under relatively low levels of automatic and deliberate constraints. Fantasies, however, are typically more constrained than daydreams. While the distinction between daydreaming and ordinary fantasizing can blur under certain conditions, it will become clear in the discussion below that *compulsive* fantasy, which is highly constrained in multiple ways, is incompatible with daydreaming.

Imagination is a heterogeneous category (Dokic & Arcangeli, 2014; Kind, 2013) connected to memory and visualization, supposition and artistic engagement, creative production and problem-solving, and many other mental experiences. In this paper, we understand imagination as imagery-involving mental experience that is also speculative: we can imagine events, scenes, or objects without any commitment to whether or not they actually exist (Thompson, 2007; Martin, 2002). We can, for example, imagine possibilities, distant realities, or fictions. Mental imagery can be more or less vivid and can include imagery in exteroceptive sensory modalities (e.g., somatic, auditory, or olfactory), as well as interoceptive sensory modalities (e.g., kinesthetic, nociceptive, or visceral). To imagine in a sensory way is to simulate perceptual experience. Such experiences take many forms, including inner speech, a sense of presence, or a simulated bodily feeling (Thompson, 2007, 2015).

Daydreaming and fantasy are both imaginative activities that share an *immersive* quality, meaning that sensory imagination is accompanied by a feeling of spatiotemporal presence, in fitting with the usage of the term “immersive” in dream research (Windt, 2010). This sense of presence can centre on a vantage point internal to a participant in a scene or an observer of the scene. That is, immersive presence connotes, at a minimum, a viewpoint on, or experience of, an unfolding spatiotemporal construct. Not all imagination feels immersive—we may experience fleeting mental images, imagistic thought, inner speech, or counterfactual supposition without this sense of experiential presence. Immersive imagination occurs in a wide range of contexts, from reading fiction to meditative visualization.

Fantasy has several characteristic features. Importantly, fantasies are emotional and narrative driven. Fantasizers also typically identify with a virtual proxy and can derive emotional gratification through imagining the proxy’s experiences (Butler, 2006; Casey, 2003). A romantic fantasy, for example, might gratify through the imaginative experience of having one’s affections returned; a revenge fantasy might gratify through the imaginative experience of seeing a bully receive their comeuppance. An imagined proxy can be a version of the fantasizer’s self, such as an idealized self, or someone other than the self. Compulsive fantasizers often describe imagining idealized versions of themselves. The following quotation from a self-described MDer sharing their experiences on a Reddit forum illustrates this identification with an imaginary, idealized self, typical of fantasy (see Appendix A for quote selection methodology):

“I have my own world in my head where I am a charismatic beautiful super model of a different race who can get anyone they want romantically. I have many genuine friends and make them easily. I am wealthy, intelligent, successful, happy.”

(midnightmoodaway, 2025)

Fantasies can play out on any theme, but narrativity, emotional gratification, and identification can distinguish fantasies from other immersive imaginings. For example, one can immersively imagine how an audition might go without fantasizing about it. However, the same scene will become a fantasy if the imaginer relishes the gratifying imaginative experience of astonishing the judges and becoming a star, for example. Daydreams can share content with fantasies and therefore cannot be distinguished based on thought contents alone. However, fantasizing and daydreaming characteristically differ in terms of the constraints that shape their dynamic unfolding.

An important way to distinguish fantasy from daydreaming has to do with their differing levels of deliberate constraint. Typical fantasies, unlike daydreams, are agential, unified imaginative projects. Crafting a fantasy narrative is often a purposeful activity. (Butler, 2006, p. 48). In contrast, daydreaming feels aimless and arises in the associative flow of spontaneous thought (Lawson & Thompson, 2024). Spontaneous thought here should be understood as “a mental state, or series of mental states, that arise relatively freely due to an absence of strong constraints on the contents of each state and on the transitions from one mental state to another” (Christoff et al., 2016, p. 719). The spontaneous imaginative episodes that constitute daydreams arise associatively in a relatively unconstrained, easeful way (Stan & Christoff, 2018). Both daydreaming and mind-wandering have this easeful quality; however, daydreaming also involves immersive imagination, whileereas mind-wandering does not (see Lawson & Thompson, 2024). Unlike daydreams, which generally feel aimless, prototypical fantasies are marked by higher levels of deliberate constraints in the form of intermittent cognitive control guiding the unfolding of narrative contents. Again, daydreaming and fantasy should be understood as existing on a spectrum, and it is possible to begin fantasizing spontaneously. Many fantasies, however, are imaginative creations undertaken or developed purposefully.

Because they evoke emotions and centre on unified, often long-form narratives, fantasies also can operate under higher levels of automatic constraints than typical daydreams.^2^ One’s emotional involvement in narratives and imaginary worlds in fantasy often contributes to increased affective salience: strong emotion can exert a pull on thought. The pleasure of emotional gratification also commands cognitive resources in an automatic way. Narrative guidance, discussed in detail below in the context of compulsive fantasy, also acts on fantasies more generally in the sense that imagined elements and narrative developments internal to the imagined story or the imagined world itself can propel imaginative episodes in specific directions. Fantasy narratives, compulsive or otherwise, may additionally be “extended” over long periods and over many episodes of fantasizing (Bruineberg & Fabry, 2022), which can increase narrative complexity as well as emotional attachment to characters. Even when they are not deliberately initiated, fantasies feel purposeful and constrained, not aimless and idle as daydreams typically do.

Daydreams and fantasies also differ in terms of their levels of perceptual decoupling. While daydreams are usually characterized by loose attention and loose connections between imaginative episodes, fantasies tend to hold attention rapt, resulting in unified, extended, coherent episodes. Fantasies are often colloquially described as escapist, perhaps because absorption in fantasy may be accompanied by higher levels of perceptual decoupling from interoceptive and exteroceptive sensations. In part because of their compelling narratives, fantasies often demand more mental bandwidth and executive processes, making fantasies feel more absorbing. Because daydreamers easily flow in and out of daydreams, often without being explicitly attentive to the fact that they are doing so, it is often easy to engage in activities like driving or washing dishes while daydreaming. Daydreams usually end without the imaginer’s notice when other thoughts or tasks arise. Fantasy, however, requires more mental bandwidth and because of that, it can be harder to disengage from fantasies, especially as automatic constraints increase in the context of *compulsive* fantasy. As suggested by the following quotation, some immersive fantasizers describe the virtual worlds of their fantasies as inhibiting their engagement with the external environment to the point that they are concerned they may not be able to attend to daily activities simultaneously:

“I am really scared that I may start daydreaming wile driving especially when I am alone I haven’t driven alone yet but I am scared that I will get lost in my thoughts and get into accident”

(Top-Cat6153, 2025)^3^

Especially when they are crafted by skilled imaginers (Kind, 2020), immersive fantasies can compete with everyday life and monopolize cognitive resources.

Under conditions we can now specify, a daydream can sometimes blur into a fantasy, or a fantasy can blur into a daydream, on a spectrum of imaginative activity. An aimless imagining might unfold in the stream of mind-wandering but develop into an emotionally gratifying narrative, for example. If the imaginer begins to evaluate and deliberately craft the story towards an emotionally gratifying end, this immersive imagining will become a fantasy. Alternatively, a gripping fantasy might loosen its hold, easing associatively into daydreaming or mind-wandering as constraints decrease. Though daydreams and fantasies are not strictly incompatible, they are distinct phenomena. By virtue of their low constraints, daydreams feel aimless, and they hold attention more loosely, even if they can sometimes share other features with fantasy. Fantasy imagination is more constrained, typically feels more purposeful, and often monopolizes attention in addition to involving narrative, emotional gratification, and identification.

When fantasy becomes compulsive, it diverges more radically from daydreaming. Fantasies that exert an addictive pull typically become highly constrained. Compulsive fantasies, as we will see, are not free-flowing, spontaneous mental experiences. In other words, compulsive fantasies are not daydreams. The term “daydreaming” in “maladaptive daydreaming” potentially obscures the relatively high degrees of deliberate and automatic constraints on thought that govern this distinctive mental activity. The dynamics of compulsive fantasy are complex and governed by oscillations and tensions between automatic constraints and deliberate constraints. In the next two sections, we turn to the role of automatic (Section 3) and deliberate (Section 4) constraints on thought and their variations in the emergence of episodes of compulsive fantasy within the structure of the DFT.

## 3. Automatic Constraints During Compulsive Fantasy Onset

Compulsive fantasy is often characterized by a struggle with agency. Compulsive fantasizers often feel unable to control their urges to fantasize; at the same time, many report that fantasy activity regulates emotion in part because crafting fantasies is a source of agency, and fantasy contents are not subject to the limitations and struggles that inhibit agency in the imaginers’ everyday lives. This tension between lack of felt agency and regaining agency through imagination characterizes the struggle of compulsive fantasy, and the remainder of this paper will expand on this. Compulsive fantasy is a complex process, and the constraints that govern the *onset* of compulsive fantasy episodes differ from the constraints governing their unfolding. In this section, we discuss the role of automatic constraints in fantasy onset.

Compulsion is a paradigmatic form of automatic constraint. Phenomenologically, the feeling of compulsion would manifest as being pulled towards engaging in fantasy. Compulsive fantasizers often yield to urges to begin fantasizing despite conflicting preferences to do otherwise (Bigelsen et al., 2016; Somer et al., 2016a). The strong pull to begin fantasizing likely reflects relatively high levels of automatic constraints limiting the range of potential thoughts.

The main types of automatic constraints on thought discussed in the literature to date include affective salience, sensory salience, and mental habits (Christoff, 2024; Christoff et al., 2016). Affective salience appears to be a particularly strong source of automatic constraint in compulsive fantasy (Somer, 2002; Wen et al., 2024). Affective salience is defined as “the tendency of one aspect of experience to stand out due to its association with emotional arousal” (Christoff, 2024, p. 397). Emotional experiences in the real world may restrict thought, and thoughts may be automatically constrained in order to engage with or regulate emotions. As one self-identified MDer states:

“I’ll often find that I’m subconsciously drawn to a scene where one of the characters is feeling an emotion that I’m feeling in real life. I think it’s my mind’s way of processing emotions”

(Diamond_Verneshot, 2025)

Through engaging with fantasy related to ongoing emotions, compulsive fantasizers may find a safe place to cope with these emotions and achieve a sense of agency through active participation in a storyline–something they might not otherwise be able to achieve in everyday life. There is evidence that processing emotions in this way may be beneficial for emotional regulation skills and feelings of social connectedness (Poerio et al., 2015, 2016). Those who experience less agency in the real world, and especially those who struggle to manage traumatic experiences or extreme loneliness, may be more susceptible to relying on compulsive fantasy for emotional regulation. Traumatic experiences in childhood and social anxiety have been identified as potential predictor for the development of compulsive fantasy (Somer & Herscu, 2017). Compulsive fantasizers often report that real-world negative emotions prompt them to begin fantasizing as a form of escape or coping (Somer, 2002; Wen et al., 2024). The affective salience of a situation may prompt an emotion-regulating fantasy, perhaps through a storyline that simulates satisfaction or control unattainable in everyday life. As compulsive fantasy is often used to cope with difficult realities, it is likely that affective salience is a primary automatic constraint at the onset of compulsive fantasy episodes.

Habit-based thought patterns may also act as automatic constraints (Christoff, 2024) on the onset of compulsive fantasy. The development of fantasy from a frequent behaviour to a potential disorder may be driven by automated thought patterns. As individuals fantasize more regularly, repeated relief from negative emotions may reinforce this mental behaviour, making those mental states more easily accessible in the future. For example, if someone frequently engages in fantasy after experiencing a negative emotional event, the next occurrence of a negative event may become more likely to prompt the desire or pull towards fantasizing. This is demonstrated by the following quotation:

“my brain has sort of been trained to escape into daydreams whenever I’m stressed, bored, or overwhelmed”

(*I Thought for a Long Time I Had Maladaptive Daydreaming, but I’m Still Not Sure*, 2025)

Even when this form of relief or escape is not initially sought compulsively, over time and repeated experiences, the process of fantasizing to emotionally regulate can become habitual, strengthening feelings of compulsion towards engaging in fantasy due to the additional automatic constraints of habit (Todd et al., 2012). Due to these heightened automatic constraints, initially easeful and freely chosen fantasizing can, in time, become compulsive. This strengthening of automatic constraints may be similar to the reinforcement that occurs in addictions. As one Reddit user self-identifying as a MDer attests:

“It’s not just a habit but also an addiction and automatic brain response”

(Tiny-Supermarket5036, 2025)

In fact, compulsive fantasy has been conceptualized as a behavioural addiction due to its compulsive nature and detrimental effects (Pietkiewicz et al., 2018; Somer & Herscu, 2017). In the context of addiction, cravings are highly automatized forms of thought that arise in response to cues, environments, or behaviours associated with the addiction (Burrell et al., 2025; Renaud et al., 2021). Like cravings, urges to engage in compulsive fantasy are often triggered by music, media, or real-world situations (Bigelsen & Schupak, 2011), as exemplified by the following quotation from a self-identified MDer:

“Everything is a trigger. TV, music, even looking at myself in the mirror.”

(Unlucky_Trash1685, 2025)

As fantasy sessions become more regular, habitual compulsions may strengthen and detrimental effects may appear. Further engaging with the behavioural addiction may exacerbate the cycle: fantasizing comes to occupy an average of 56% of waking hours for compulsive fantasizers (Bigelsen et al., 2016; Bigelsen & Schupak, 2011), taking the place of real-world social interaction and the pursuit of other life projects. This may, in turn, increase feelings of loneliness and helplessness, generating negative affect, which can trigger more powerful and frequent urges to fantasize. In fact, compulsive fantasizers reported significantly stronger urges to return to fantasizing after being interrupted, compared to control participants (Bigelsen et al., 2016; Bigelsen & Schupak, 2011). This demonstrates the automatic constraints that may be increased by the habitual process of engaging in compulsive fantasy.

In addition to affective salience and habitual mental states, sensory salience has also been characterized as a prominent form of automatic constraint on thought. Sensory salience is defined as the “tendency of one aspect of current perceptual experience to stand out relative to other aspects of perceptual experience due to its sensory features, such as high perceptual contrast” (Christoff, 2024, p. 397; see also Anderson, 2019). As we show in the following section, compulsive fantasizers often deliberately reduce the salience of their sensory environment in order to increase their immersion into fantasy. This does not mean that sensory salience cannot play a role in fantasy onset, however. As we saw above, triggers to fantasize can be various, and sensory triggers may be among them. In particular, distress related to salient sensory experiences may trigger the onset of compulsive responses, perhaps by prompting emotionally regulating fantasy rituals. For example, a study of 510 people found that 49.8% of compulsive fantasizers claimed to experience obsessions with being “bothered by certain sounds or noises,” and 48.8% reported somatic obsessions around a “body part or aspect of appearance” (Salomon-Small et al., 2021, p. 348). Strong affective salience of sensory experiences, especially those of a negative nature, may cue affective changes and consequent urges to fantasize. However, the urge to fantasize is likely more driven by affective salience than by sensory salience, even when sensory triggers are involved.

Automatic constraints, particularly those related to affective salience and habits, may combine to create strong urges to fantasize that can become compulsive over time. Given the presence of these strong automatic constraints, compulsive fantasy cannot be equated with forms of spontaneous thought such as daydreaming or mind-wandering, which are marked by relatively weak automatic constraints (Christoff et al., 2016; Lawson & Thompson, 2024). The automaticity of compulsive fantasy may make it seem as though there is nothing deliberate about the activity. As we will see in the next section, however, this is not the case.

## 4. Deliberate Constraints During Compulsive Fantasy Onset

Deliberate choices to attend to thoughts or features of one’s environment can limit the breadth of potential thoughts and ideas, at least temporarily. In addition to being automatically constrained, compulsive fantasies are also often deliberately constrained. Compulsive fantasizers often choose to begin fantasizing and shape the conditions to facilitate immersion prior to onset. This can occur in the context of conflicting desires to do otherwise. Bigelsen and Shupak write that the “inability to control the fantasizing does not mean that the fantasies always appear effortlessly or unwillingly. Instead, even though the fantasies are often consciously brought forth, participants reported being unable to control their desire to create the fantasies” (Bigelsen & Shupak, 2011, p. 1644). This suggests that the subjective feeling of control is not necessarily the same as the process of exerting executive control. That some fantasies are consciously brought forth is illustrated by the following quotation from a Reddit user self-identifying as a MDer:

“Most of my daydreaming episodes comes with the conscious decision and willingness to take part in these daydreams”

(Dazzling-Ad3857, 2025)

Compulsive fantasizers often report feeling a sense of ownership over their creations and intentionally situating themselves in particular environments to promote emotional engagement and immersion (Somer et al., 2016b).

Compulsive fantasizers often deliberately control the onset of their fantasy experience in a variety of ways. This may be accomplished by setting the stage for a scene to unfold in imagination. For example, a fantasizer might methodically bring features of a virtual environment into awareness. Fantasizers may also deliberately manipulate conditions in their external environment to be conducive to fantasizing, such as isolating themselves from other people. Fantasizers may also deliberately facilitate high levels of immersion through perceptual decoupling, disconnecting from the external world by attenuating their sensations. Compulsive fantasizers report a wide range of decoupling tricks: covering their heads with blankets, pacing, acting out their fantasies, and listening to music are among the most commonly reported behaviours (Bigelsen et al., 2016; Bigelsen & Schupak, 2011; Somer, 2002). Many compulsive fantasizers observe that music, in particular, facilitates immersion. Some self-identified MDers find that listening to familiar music enhances their fantasies, because novel elements of unfamiliar songs can be distracting. One self-described MDer writes the following:

“I realized that most of my daydreaming happens when I’m listening to songs I’m very familiar with. when i’m listening to new music, I’m more inclined to notice the lyrics or chord progression, instead of daydreaming!”

(MoonyDropps, 2025)

Bodily movement is another particularly common strategy. In Bigelsen and Schupak’s (2011) sample of compulsive fantasizers, 79% reported engaging in repetitive kinesthetic movements during fantasy episodes. Movements such as pacing, swinging, or hand movements, may act to limit the variability of the sensory experience and serve to attenuate exteroceptive sensations (Braun et al., 2005; Levin & Benton, 1973; Tannan et al., 2005), potentially clearing the way for further immersion into the internal experience (Bigelsen et al., 2016).

At least some of these strategies seem to be deliberately employed before fantasy episodes begin. Many compulsive fantasizers isolate themselves before beginning to fantasize to avoid judgement when engaging in these behaviours, suggesting some instances of deliberate choice. Not only do deliberate choices in the moment constrain thought to the fantasy, but long-term planning and engagement also often operate in the background to make these choices effective. For example, some compulsive fantasizers and immersive daydreamers^4^ describe crafting playlists of songs relevant to their characters or storylines, or planning ahead to fantasize when no one else will be home. One self-described MDer describes their planning in the following way:

“I make the entire room dark and put my earphones and turn on whichever playlist is fitting to my current daydream.”

(Ok_Bodybuilder_8452, 2025)

The fact that planning is involved is particularly indicative that compulsive fantasizers deliberately constrain and facilitate the initiation of their fantasy behaviour to some extent. This planning can also include research, reading, and writing. Prior to the onset of any particular episode, compulsive fantasizers and immersive daydreamers alike often do preparatory research for their next venture into their fantasy, much like an author would. On online forums, self-described maladaptive daydreamers compare notes and describe illustrating their characters, seeking out various forms of media for inspiration, creating mood boards, or writing notes. One self-described MDer writes:

“I for instance can create entire buildings and cities and keep it consistent with the help of a pinterest picture as inspiration.”

(Cool_Spell_2944, 2025)

Another writes:

“im also an artist so i try to sketch out stuff the next day of the stories i came up with at night. i get so fixated on them i make pinterest boards and write for hours in google docs. sometimes i write movie scripts too”

(ttaemin, 2025)

For some, these behaviours are foundational to constructing the long-term agential projects many fantasies comprise. Immersive fantasies can unfold over years or even decades, and imaginers often become profoundly emotionally attached to their creations. For example, one Reddit user shares the following:

“After 7.5 years of being in love with my character, I’ve been wondering if I’d symbolically ‘marry’ him one day”

(DryCoast, 2025)

Due to the prominence of these deliberate behaviours occurring beyond and before particular episodes of compulsive fantasizing, we find it difficult to paint daydreaming, fantasy, and compulsive fantasy with the same brush. There appears to be a large discrepancy between the easeful, spontaneous arising of daydreams and the high levels of deliberate guidance described by compulsive fantasizers.

At the same time, as discussed above, fantasies can sometimes begin without explicit intention. Another Reddit user describes two different trajectories of fantasy onset:

“I think most of us here have that intentional type, where you lock yourself in a room and start creating stories, moving your hands, walking in circles, etc. But I also realized that I have another type of daydreaming that happens very quickly and is completely out of my control, like in the movie The Secret Life of Walter Mitty. Something happens in front of me, and I automatically imagine the situation in a completely different way. Then, I “wake up” to reality.”

(Key-External-9122, 2025)

Compulsive fantasy is not simply an automatic behaviour or a deliberately chosen behaviour; we propose that the interplay between deliberate and automatic constraints guides the onset of compulsive fantasy. Episode onset may often occur when automatic constraints exert a pull to fantasize that is realized in part by mental and physical actions under deliberate guidance. This often occurs in tension with desires or deliberate efforts not to fantasize. Prior to the fantasy event, strong automatic constraints may drive the urge to engage in fantasy. This could be due to the affective salience of a real-world event (e.g., a negative social media post in one’s feed) or the desire to escape reality, which may precipitate the compulsion to fantasize. During this pre-fantasy period, deliberate constraints may increase in order to combat the “relentless pull of [fantasizers] imagination” (Bigelsen & Kelley, 2015). On the one hand, deliberate constraints may potentially override the automatic constraints initiating a fantasy episode and prevent it from beginning. On the other hand, however, compulsive fantasizers may use deliberate constraints, for example, to establish the scene. The interplay of constraints characterizing the *onset* of compulsive fantasy is distinct from the dynamics of automatic and deliberate constraints that govern the *unfolding* of thoughts within a fantasy. In the next section, we discuss how the dynamics of automatic and deliberate constraints facilitate the unfolding of fantasy.

## 5. Dynamics of Thought During the Unfolding of Compulsive Fantasies

**Figure 2:**
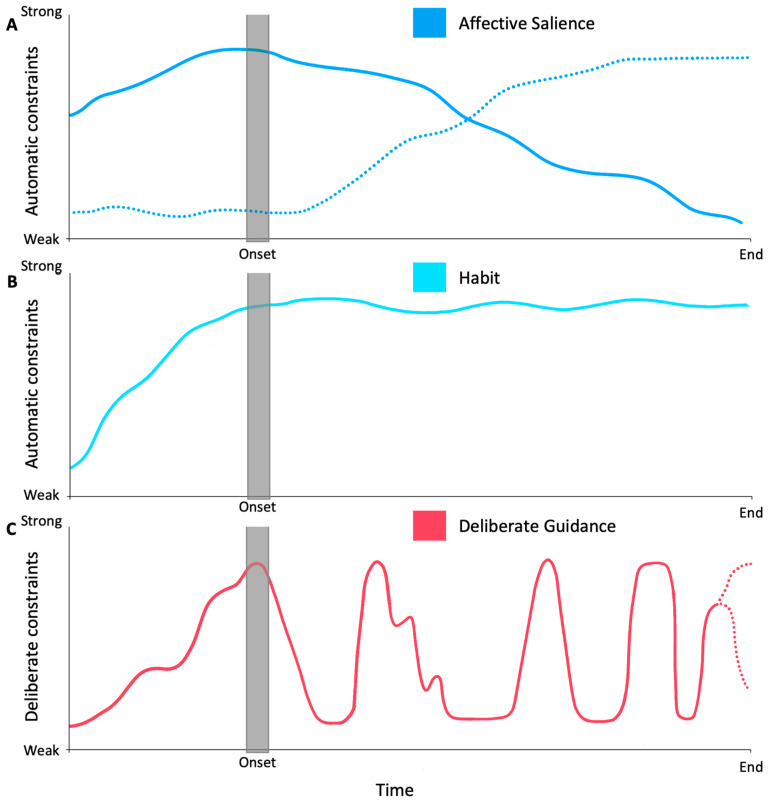
An example analysis of the proposed dynamics of constraints during the onset and unfolding of a compulsive fantasy episode. In this example, automatic constraints, such as affective salience (**A**) and habitual patterns of thought (**B**), create urges to initiate a fantasy episode and shape its unfolding. Automatic constraints, such as dysphoria or distress, initiate an episode and then decline during its unfolding (solid line in **A**). Other automatic constraints, such as emotional salience due to gratification, increase during the episode and influence its progress (dotted line in **A**). Deliberate constraints (**C**) are intermittently engaged to flexibly direct the fantasy. As some forms of affective salience weaken, strengthening deliberate constraints may precipitate the end of the fantasy (rising dotted line in **C**). Alternatively, the end of the fantasy may be brought about by other automatic constraints not pictured here, accompanied by a decrease in deliberate constraints (descending dotted line in **C**). The portrait dynamics are not meant to be representative of all compulsive fantasy episodes but are instead intended to represent possible dynamics. Different trajectories, especially at the end of an episode, are also possible.

Once the fantasy episode has been initiated, a dynamic interplay between automatic and deliberate constraints shapes the fantasy throughout its duration. This interplay can help to explain reported experiences: for example, compulsive fantasizers often report letting their ‘mind take control’ and experiencing their fantasy as if it were a movie or TV show (Bigelsen & Kelley, 2015). This phenomenological description may reflect the oscillations of constraints on thought that occur across the episode of compulsive fantasy (Figure 2).

Following the onset of a fantasy, deliberate constraints may temporarily weaken, resulting in the phenomenological experience some compulsive fantasizers describe of seeing where the mind goes. One writes:

“I made a bunch of “seeds”, places, events, monsters, people. And I try to put my mind into a random person there and let it run crazy with story creation. It’s not structured, it’s hard to feel like I have control over it, but it feels like the best way to really use my daydreaming to it’s full creativity.”

(Key-Acanthocephala10, 2025)

Once initiated, fantasies may unfold with relatively little deliberate guidance, guided instead by automatic constraints, such as affective salience (Figure 2A), habitual mental patterns (Figure 2B), and societal or narrative constraints (not pictured on Figure 2). In the context of an unfolding imagined story, these automatic constraints can limit the option space for the narrative’s progression. For example, an imaginer’s mood may affectively guide a narrative’s direction; a foul mood, for example, might suggest more negative fantasy content. The narratives of fantasies may also, reciprocally, shape affect and increase emotional salience. Habits formed through repeated fantasizing may suggest familiar narrative pathways. Similarly, an imaginer’s societal context may constrain the kinds of stories that seem available or appropriate. These automatic constraints likely guide the episode without necessitating a feeling of deliberate guidance.

Although various automatic constraints may remain relatively strong across the episode, the strength of deliberate constraints likely fluctuates over the course of the fantasy episode (Figure 2C). Periods of automatically guided fantasy may be punctuated by explicit interventions on the part of the imaginer. Compulsive fantasizers often report re-engaging deliberate control over fantasy content in a variety of contexts. They may deliberately intervene to shape the fantasy narrative when they want, for example, to revise a plot that has taken a wrong turn, when they wish to replay a scene, or when decisions must be made on behalf of characters.

Sometimes, automatic constraints and deliberate control are experienced as being in tension. Tensions can arise when fantasies seem to go awry. As the following quotation illustrates, some fantasizers report having to “correct” cases of “misbehaving imagery”:

“Recently I’ve been struggling with misbehaving imagery. It feels like my mind fixates on one thing and struggles to switch, like if a character is dressed and then undresses my brain will struggle between them having fabric on and not, back and forth. It’s a conscious thing to try and correct.”

(xxangelbunnyxx, 2024)

This quotation demonstrates the broader struggle with agency that often characterizes compulsive fantasy. In this example, a struggle with automatic constraints pervades the fantasy itself. This struggle manifests under high levels of automatic constraints governing fantasy, countered by strong deliberate constraints. Deliberate fantasy engagement appears to be a way for compulsive fantasizers to exercise control despite compulsive fantasy feeling largely uncontrollable. The strength of deliberate constraints may oscillate across the duration of a fantasy episode, exerting a strong influence on the progression of the fantasy when engaged, and then relaxing to allow for the narrative to progress through automatic guidance.

In fantasies, like in other imaginative projects such as novel-writing, automatic constraints intrinsic to the contents of imagination can shape the project without the conscious intervention of the imaginer. For instance, a well-known character’s established personality traits will suggest a delimited range of fitting actions; imagining a scene that takes place in a meadow will suggest background elements like wildflowers and dragonflies, not jellyfish or racecars. In this sense, contextual constraints are usually at work even in imagined narratives that span only one episode. However, they can increase as imaginings become more elaborate, especially with respect to long-term imaginative projects. As fantasies persist and repeat, many compulsive fantasizers and immersive daydreamers alike report developing cohesive storylines that persist across episodes lasting months, years, or even decades. As imagined contents build on one another in the context of cohesive worlds or narratives, new contents added to the schema of the fantasy constrain and determine what further events fit the narrative or world. In this way, automatic narrative constraints compound: each new property of the imagined world produces additional automatic constraints that will influence the dynamics of thought within the fantasy episode and across future episodes—at least when narratives are coherent and internally consistent. Hence, while some automatic constraints loosen as fantasies unfold, other automatic constraints may increase.

Fluctuations in automatic constraints operate at both short and long timescales. Automatic constraints relating to the experience of compulsion and affect that originally drove the onset of the episode may develop quickly and be slowly mediated by engagement with the fantasy. This is supported by evidence from self-reports and empirical findings suggesting that engaging in forms of social thought, such as fantasy, can enhance mood and feelings of connection (Poerio et al., 2015, 2016). These benefits may reduce the salience of the initial affective constraints on thought across the fantasy episode. However, the original feelings of distress or loneliness may be replaced with feelings of satisfaction or pleasure, that may, in turn, further propagate the fantasy episode. These alterations in automatic constraints may operate at a much longer timescale than intermittently recruited deliberate constraints. Pleasure motivates continued engagement: many self-identified MDers describe their fantasies as almost endlessly compelling. As one compulsive fantasizer attests:

“Any chance I had, I’d go and lie down or I’d just get sucked into it, and it was so hard to turn off. It’s like you’ve always got a tailor-made fantasy, soap opera, action film, whatever you want, playing in your head all the time. An alcoholic can run out of booze and money, but you don’t run out of mind. You can’t just tell yourself to stop thinking.”

(Tsoulis-Reay, 2016)

This raises the question of how fantasy episodes conclude. If fantasies are so gratifying and propulsive, how do constraints change to allow disengagement?

For some, the loosening of initial automatic constraints relating to craving may facilitate the end of the episode, as the subsiding of an emotionally upsetting feeling may precipitate a feeling of satisfaction and a natural end to the fantasy. In other words, the automatic constraints that may have driven the onset of the event may gradually decrease as they become satisfied over the course of the fantasy. However, for compulsive fantasizers, a decrease in some automatic constraints, such as affective salience, may be overshadowed by habit-based automatic constraints, which may make disengaging from the fantasy difficult. One compulsive fantasizer describes this difficulty in vivid terms:

“I need to brush my teeth before sleeping right? I can’t bring myself to do so since I’m busy pacing around and daydreaming—but I also can’t sleep unless I brush my teeth. I go by the sink thinking “I will brush my teeth now” then I turn around and keep pacing and daydreaming again. This goes on for 2–3 h on average, and I end up sleeping very late.”

(Few-Vegetable-7108, 2025)

In this case, the end of an episode appears to be brought about by strong deliberate constraints forcing thoughts away from the fantasy, or perhaps by strong external pressures contradicting the habitual constraints, such as societal pressures to maintain employment or social relationships. Other automatic constraints on thought may break absorption in the fantasy: for example, the sensory salience of being interrupted by something in the external environment, or the affective salience of feeling shame for wasting time, may be strong enough to end a fantasy episode.

## 6. The Relationship Between Creative Thought and Compulsive Fantasy

On the analysis of compulsive fantasy developed above, compulsive fantasizers do not just witness internal episodes that unfold outside of their control, but also intentionally construct their fantasies through implementing a range of deliberate constraints. The oscillations of deliberate constraints in the context of an overall guidance by automatic constraints during the unfolding of imaginative fantasies, including the compulsive fantasies we discuss here, resemble the dynamics proposed to facilitate creative thought. Creative thought, understood as a dynamic phenomenon, is believed to consist of two alternating processes: the generation phase, during which there are relatively few deliberate constraints and ideas arise spontaneously; and the evaluation phase, during which deliberate and automatic constraints are used to consider the ideas for appropriateness (Christoff et al., 2016; Ellamil et al., 2012; Girn et al., 2020; Zamani et al., 2022). Episodes of creative thought processes and compulsive fantasy episodes may both be initiated during periods of high automatic and deliberate constraints, followed by the reduction of deliberate constraints to facilitate the development of ideas that are then evaluated.

The evaluation phase of creativity is characterized by heightened constraints. Automatic constraints have been proposed to increase as the period of creative thought goes on (Girn et al., 2020). This differs from our conceptualization of the alterations of the distinctive automatic constraints that occur during a typical compulsive fantasy episode, which likely decline over the course of an episode as the activity relieves pressures, such as strong negative affect. This difference may be a productive area of future research. However, we propose that other automatic constraints, such as mood, social norms, habitual patterns, or narratives, also shape compulsive fantasy narratives throughout the course of an episode.

This interplay between deliberate and automatic constraints on thought suggests a conceptual overlap between creative thought and compulsive fantasy. This overlap is particularly clear in the phenomenology of the two forms of thought. Like self-reports of creative thought, self-reports of compulsive fantasy often include a feeling of letting go of control to allow the mind to be free, to see what comes up or where the mind goes.

A connection between compulsive fantasy and creativity has been under investigation in recent empirical work (Thomson & Jaque, 2023; West & Somer, 2020; Zedelius et al., 2021). However, the majority of research to date has not utilized objective measures of creativity with compulsive fantasizers. For example, one study of creativity in compulsive fantasizers (West & Somer, 2020) uses the Biographical Inventory of Creative Behaviours, which scores creativity based on subjective reports of past engagement in creative behaviours, such as “writing a short story” or “producing a short film” over the last 12 months (Batey, 2007). This study found a negative relationship between creativity and compulsive fantasy (West & Somer, 2020). However, this focus on creative products, rather than creative thought processes, may be misleading. Since compulsive fantasizers may spend over 55% of their waking time engaging in fantasy (Bigelsen et al., 2016; Bigelsen & Schupak, 2011; Somer et al., 2016a), their overall output of creative products like short films may be lower than a control group because they may be engrossed by their fantasies, and fantasizing is not encompassed by the questionnaire as a potentially creative experience in its own right. For this reason, an apparent negative relationship between creativity and compulsive fantasy may be due to the use of measurements focusing on creative products rather than measures of creative capacities or dispositions, creative thought processes, or mental creations.

Similarly, Thomson and Jaque’s (2023) study on compulsive fantasizers reported mixed findings while using two questionnaires to measure creativity: the Experience of Creativity Questionnaire, designed to implement qualitative methods to assess the phenomenology of creativity (Nelson & Rawlings, 2009), and the Short Scale of Creative Self which captures measures of “creative personal identity” and “creative self-efficacy” (Karwowski et al., 2018). “Creative personal identity” is defined as the “extent to which creativity is treated as an important part of an individual’s identity” and “creative self-efficacy” as the “conviction about one’s own capabilities to manage creative challenges” (Karwowski et al., 2018). In this study, creativity was understood as a personality trait measured by scores indicating how much participants related to statements such as “My creativity is important to who I am” or “I trust my creative abilities” (Karwowski et al., 2018). To our knowledge, no study to date has investigated the connection between creativity and compulsive fantasy using measures of creativity that encompass imaginative activities irrespective of the products they result in.

The measurement of creativity by frequency of engagement in creative behaviours is also found in studies of daydreaming, understood as spontaneous thought unrelated to one’s current situation (e.g., Sun et al., 2021). One study on daydreaming—defined as “thought unrelated to the here and now,” did use objective measures of creativity and reported that personally meaningful daydreaming predicted self-reported creative behaviour, and fantastical daydreaming predicted creative writing quality and day-to-day creative behaviour (Zedelius et al., 2021, p. 1). The findings of this study suggest that fantasy, which is frequently reported to be personally meaningful and fantastical by the authors’ definitions, is closely related to the process of creative thought or is itself a form of creative thought. Future investigations into compulsive fantasy and creativity are needed to examine the underlying processes involved in the production and progression of these forms of thought, in order to better understand the similarities and differences in dynamics of automatic and deliberate constraints during these mental phenomena.

## 7. Conclusions

Fantasy and daydreaming are distinct imaginative phenomena, and exploring the distinction between them is essential to appropriately conceptualizing and investigating compulsive fantasy. The dynamic account we have provided here can help to clarify how various automatic and deliberate constraints govern the onset and dynamic unfolding of compulsive fantasy episodes. Clearly conceptualizing and distinguishing different imaginative activities is needed to ensure rigor and clarity in evolving research programmes targeting mental experiences, including compulsive fantasy. On our analysis, fantasy is associated with a higher degree of deliberate constraints than daydreaming and mind-wandering. This supports the use of the term “compulsive fantasy” which better captures the phenomenon of interest than the term “maladaptive daydreaming.”

Our proposed dynamic account of compulsive fantasy also sheds light on the ways that compulsive fantasy relates to agency and its implications for wellbeing. According to many reports, compulsive fantasy is often triggered by feelings of reduced agency in everyday life (Somer, 2002; Somer & Herscu, 2017; Wen et al., 2024). Engagement with compulsive fantasy may create feelings of agency in the inner world—feelings that may not be accessible through engagement with the material and social conditions of everyday life. At the same time, the compulsive urge to fantasize can also undermine one’s agency in everyday life, affecting work and relationships. This may perpetuate a cycle whereby compulsive fantasizers cede agency in everyday life, while reinforcing the automaticity that makes their engagement more habitual and eventually addictive. The more compulsive the activity becomes, the more it may interfere with real life and the more reason there may be to escape from life into compulsive fantasies. Analyzing the experience of compulsive fantasy in a way that distinguishes the onset of the activity from its unfolding can make sense of this. High automatic constraints prior to onset can contribute to a seemingly uncontrollable urge to fantasize, which undermines agency. However, in the process of fantasizing, the fantasizer may find not only emotional gratification in the fantasy’s engrossing narrative, but also a sense of agency in authoring the imagining. This dynamic account can help explain why a struggle with agency may contribute to distress.

Complex tensions within the experience of agency also play out as fantasy episodes unfold. As we have shown, the onset of compulsive fantasy is guided by both automatic and deliberate constraints. Affect and habits constrain thought and create the compulsion to engage in fantasy. Deliberate constraints then operate in a number of potential ways, such as directing thoughts away to prevent the onset of an event or deliberately guiding behaviour to enhance dissociation and engagement with the fantasy. After the onset of a compulsive fantasy, deliberate constraints are flexibly engaged to impart agency over the ongoing narrative. At the same time, the automatic constraints that create the urge to fantasize likelymay begin to decrease as emotional regulation is achieved. Other automatic constraints, such as narrative and societal constraints, are generated by the content of the fantasy and likelymay increase across sessions as particular imagined narratives and words are elaborated. The process of imagining fantasies, which is characterized by flexible alterations of deliberate constraints and slow, relatively stable pressures of automatic constraints, is very similar to the previously proposed constraint dynamics operating during creative thought (Christoff et al., 2016; Ellamil et al., 2012; Girn et al., 2020; Zamani et al., 2022). This is perhaps indicative of an intimate connection between the underlying processes that facilitate fantasy and creativity, suggesting that creative processes are involved in generating fantasies. However, the nature of this connection has yet to be directly investigated by empirical research.

The present analysis has a number of limitations. It relies heavily on the theoretical literature distinguishing different forms of thought (Christoff et al., 2018; Dorsch, 2015; Irving, 2016; Irving & Thompson, 2018; Lawson & Thompson, 2024). Empirical findings and self-report measures from compulsive fantasizers also shape our conceptual understanding of the connections between compulsive fantasy, fantasy and daydreaming and the way that episodes of compulsive fantasizing typically unfold. First-personal insights are indispensable for research into mental experiences. Choosing quotations from these self-identified MDers (see Appendix A for details) helps elucidate the imaginative processes that occur during compulsive fantasy. However, the experiences of these MDers may not be entirely representative of the features of this mental phenomenon in the population at large. Although self-report measures are necessary when researching mental phenomena with few behavioural manifestations, the reliability of these reports is unclear. Reports gathered from public online forums may have additional limitations, due to possible self-selection biases in the individuals who contribute to these forums and the impossibility of definitively confirming that these anonymous self-reports are accurate. The existing literature would be enhanced by detailed analyses of the phenomenology of compulsive fantasy and related forms of thought in more systematic observational and experimentally controlled conditions. Research of this nature could directly test the ideas put forth by our conceptualization.

We intend our proposal as a theoretical basis for future empirical research. Future studies may also benefit from using self-report measures in tandem with quantitative measures, such as neuroimaging. Investigations into the neural dynamics of these different kinds of imaginings would allow for the testing of our conceptual understanding in relation to the neural dynamics suggested to underlie deliberate and automatic constraints within the DFT. Another interesting line of future research is the similarities and differences in deliberate constraints between daydreaming and fantasy, and the extent to which parallels could be drawn to the possibly analogous relationship between dreaming and lucid dreaming. Future studies may also investigate potential moderating factors, such as individual differences in personality traits, of the relationship between compulsive fantasy and creativity.^5^ The relationship between compulsive fantasy and creativity has yet to be empirically tested in a manner that is suitable for drawing conclusions as to the creativity of fantasy activities and the creative potential of those who engage in them. Future research into these relationships may benefit from following similar methodologies to those used to investigate creativity in daydreamers from Zedelius et al. (2021), while using specific, theoretically driven definitions of the phenomena of interest. Another way to investigate creativity is to use a retroactive recall or a phenomenological interview after a fantasy episode, which would allow for more direct measurement of creativity in fantasy. Finally, given the conceptual analysis presented here, it would be useful to develop new scales for measuring compulsive fantasy that reduce the current emphasis on impairment and dysfunction.

The extension of the DFT we present here can help elucidate the ways in which compulsive fantasy can be detrimental to imaginers by providing them first with a sense of agency within their fantasy, while concurrently undermining their opportunity to increase agency in their life outside of fantasy. This will help achieve a more precise and nuanced understanding of the conditions under which compulsive fantasy can become detrimental. The source of the harm is not in the imaginative activity itself, but in the potentially addictive nature of the reward associated with the feeling of obtaining mental agency within a fantasy, which can make the compulsive fantasizer unmotivated to pursue a sense of agency in their life outside of fantasy. Nevertheless, our analysis suggests that crafting fantasy narratives and worlds, even when carried out compulsively, may be a creative activity and a source of empowerment. We hope that the distinctions in this paper will motivate future research and contribute to improving our understanding of compulsive fantasy, with all of its richness and implications for everyday life.

## Data Availability

The data presented in this study are available in r/MaladaptiveDreaming at https://www.reddit.com/r/MaladaptiveDreaming/ (accessed on 22 September 2025) and in r/ImmersiveDaydreaming at https://www.reddit.com/r/ImmersiveDaydreaming/ (accessed on 22 September 2025).

## Notes

1 Compulsive fantasy is often comorbid with obsessive–compulsive-related disorders. For a comprehensive examination of the interactions between compulsive fantasy and obsessive–compulsive-related disorders (see Salomon-Small et al., 2021).

2 For similar reasons, daydreaming, in virtue of involving immersive imagination, tends to be more automatically constrained than mind-wandering (Figure 1), though both remain forms of relatively unconstrained, spontaneous thought.

3 Though the author of this post and others included in this paper sometimes use the term “daydreaming” in their descriptions, they are describing instances of fantasy according to our conceptualization.

4 Immersive daydreamers are people who frequently engage in immersive imaginative fantasy similar to compulsive fantasizers, but for whom fantasy does not feel compulsive or cause distress.

5 We thank the reviewers for providing interesting suggestions for future research, including those for individual differences and the connections to dreaming.

## Citation Diversity Statement

Recently, there has been increasing attention to what has been identified as a persistent pattern of gender imbalance in scientific citation practices (Bertolero et al., 2020; Chatterjee & Werner, 2021; Dworkin et al., 2020; Mitchell et al., 2013). In order to increase transparency around the representation of gender identities across the authorship teams and support the progression of correcting this imbalance, the reference list of this article has been analyzed (Zhou et al., 2020), as assessed by an assumption of the gender identification of the citation’s first author/last author. We obtained the predicted gender of the first and last author of each reference by using databases that store the probability of a first name being carried by a woman (Rowson et al., 2021; Zhou et al., 2020). By this measure and excluding self-citations to the first and last authors of our current paper, our references are distributed as follows: 40.9% woman (first author)/woman (last author), 12.1% man/woman, 7.58% woman/man, and 39.4% man/man. This method is limited in that (a) names, pronouns, and social media profiles used to construct the databases may not, in every case, be indicative of gender identity and (b) it cannot account for intersex, non-binary, or transgender people. We also obtained predicted racial/ethnic category of the first and last author of each reference by databases that store the probability of a first and last name being carried by an author of colour (Ambekar et al., 2009; Chintalapati et al., 2023; Zhou et al., 2020). By this measure (and excluding self-citations), our references contain 5.0% author of colour (first)/author of colour (last), 11.6% white author/author of colour, 15.6% author of colour/white author, and 67.8% white author/white author. This method is limited in that (a) names and Florida Voter Data to make the predictions may not be indicative of racial/ethnic identity, and (b) it cannot account for Indigenous and mixed-race authors, or those who may face differential biases due to the ambiguous racialization or ethnicization of their names.

## Abbreviations

The following abbreviations are used in this manuscript:

DFT	Dynamic Framework of Thought
MDer(s)	Maladaptive Daydreamer(s)/Compulsive Fantasizer(s)

## Appendix A

### Quotations from MDers

The quotations presented in this manuscript were collected from Reddit, an online discussion forum platform. We collected the most recent 250 posts from the user-generated online community for Maladaptive Daydreaming (r/MaladaptiveDreaming; https://www.reddit.com/r/MaladaptiveDreaming/, accessed on 20 February 2025) in order to better understand the phenomenological experience of those who identified as Maladaptive Daydreamers. These posts consisted of posts published between 2 February and 20 February 2025. All posts were considered for their viability to be included in the manuscript. The quotations that were chosen to be included in the final manuscript articulated ideas or perspectives supported by others in the community or by the existing literature.
